# Lung function impairment in children post-tuberculosis treatment: a systematic review and meta-analysis

**DOI:** 10.3389/fped.2026.1753683

**Published:** 2026-04-23

**Authors:** Hanhan Wu, Yazhen Lang, Luqing Jin, Yanqin Shen, Weiyan Pu, Rihua Zhou

**Affiliations:** 1 Department of Tuberculosis, Hangzhou Red Cross Hospital, Hangzhou, Zhejiang, China; 2 Fever Clinic of Hangzhou Red Cross Hospital, Hangzhou, Zhejiang, China

**Keywords:** children, lung function, post-tuberculosis lung disease, spirometry, tuberculosis

## Abstract

**Background:**

Pulmonary tuberculosis (PTB) in childhood can result in persistent lung function impairment in adults, but data among children remain limited. This systematic review and meta-analysis aimed to summarize and update current evidence on pulmonary function outcomes after PTB treatment in children.

**Methods:**

We searched PubMed, Embase, Web of Science, and Scopus up to October 23, 2025. Eligible studies included children (≤18 years) with PTB who underwent post-treatment spirometry. Pooled mean z-scores for FEV₁, FVC, and FEV1/FVC ratio were calculated using random-effects models. For comparative studies, standardised mean differences (Hedges’ g) were derived to compare post-PTB children with healthy controls.

**Results:**

Nine studies were included. The pooled post-treatment mean z-scores indicated significant reductions in both FEV₁ (−1.51, 95% CI −2.38 to −0.64) and FVC (−1.36, 95% CI −2.60 to −0.12), while the FEV1/FVC ratio showed no significant deviation (0.04, 95% CI −1.29 to 1.37). In case-control comparisons, post-PTB children had significantly lower FEV₁ (Hedges’ g = −0.46, 95% CI −0.78 to −0.13) and FVC (Hedges’ g = −0.29, 95% CI −0.50 to −0.08) compared with controls, while the FEV1/FVC ratio (Hedges’ g = −0.32, 95% CI −0.58 to −0.06) showed only a marginal decline. Heterogeneity across analyses was moderate to high (I^2^ = 55%–98%).

**Conclusions:**

This updated systematic review and meta-analysis indicates that children who have completed PTB treatment frequently exhibit FEV₁ and FVC values below age matched reference levels, although estimates vary substantially across studies. Pooled effect sizes should be interpreted as indicative of association rather than precise estimates of magnitude. Additional prospective, microbiologically confirmed, and age-specific studies are necessary to better understand the long-term effects of childhood TB on lung development.

**Systematic Review Registration:**

https://www.crd.york.ac.uk/prospero/, PROSPERO CRD420251166413.

## Introduction

Tuberculosis (TB) continues to pose a significant global health challenge, affecting over 10 million people in 2021, including approximately 1.25 million children under 15 years of age (1). TB primarily spreads through the air, with inhalation of bacteria leading to infection. Pulmonary TB (PTB) occurs when the body's innate immune response cannot eradicate the bacteria. Although TB is treatable, it continues to cause high rates of long-term illness and death, especially in low- and middle-income countries where over 95% of pediatric cases are reported (2). The respiratory effects of TB go beyond microbiological cure, with increasing evidence showing that many survivors—both adults and children—suffer lasting lung function problems, known as post-TB lung disease (3, 4). In 2023, a comprehensive review and meta-analysis of data from 14,621 individuals revealed that 59% of adults had abnormal lung function after completing TB treatment. Among those, 22% exhibited obstructive patterns, 23% had restrictive impairments, and 15% showed mixed patterns (5).

While the post-TB lung condition is well documented in adults, the extent and nature of these sequelae in children and adolescents are less well understood. Pediatric TB differs from adult TB not only in immune response and pathophysiology but also in its potential to interfere with ongoing lung development (2, 6). In young children, TB generally presents as intrathoracic lymphadenopathy or disseminated disease, while adolescents more frequently show adult-like parenchymal or cavitary lesions with higher bacillary loads and fibrosis (2, 6). Since lung growth continues into late adolescence, TB infection during this crucial period could impair maximal lung development, resulting in lifelong reductions in respiratory capacity (4).

Recent data show that despite successful treatment, children and adolescents still face notable respiratory issues (4). There is a reduction in tidal volume and peak tidal expiratory flow in children under 5 years old. Additionally, abnormal lung function is observed in 40% of children aged 5–10 years and in up to 65% of adolescents aged 10 years and older (7). These issues are associated with ongoing respiratory symptoms, reduced exercise capacity, and decreased quality of life. This aligns with the increasing awareness that respiratory injuries early in life, such as lower respiratory tract infections, can lead to long-lasting disruptions in lung development. This disruption can hinder recovery to pre-illness lung function levels and increase the risk of developing chronic respiratory diseases later in life (8, 9).

Despite this emerging evidence, children and adolescents remain markedly underrepresented in TB research. A recent synthesis showed that over 80% of studies on TB-associated respiratory morbidity excluded participants below 19 years of age (7). This knowledge gap limits understanding of the age-specific mechanisms and trajectories of post-TB lung disease. Previously, Lew et al (10) have also examined the effect of PTB on lung function after therapy completion, but included only five studies. Furthermore, their study did not compare TB patient data with controls due to the limited studies. Considering these limitations and the publication of several new paediatric cohort studies since this last meta-analysis, an updated review is necessary. This article, therefore, aims to provide updated evidence on pulmonary function outcomes following TB treatment in children.

## Material and methods

The protocol for this systematic review and meta-analysis was registered prospectively in the PROSPERO database (CRD420251166413). The review's conduct and reporting strictly follow the latest PRISMA 2020 guidelines (11).

### Search strategy

Two reviewers independently conducted a comprehensive literature search across PubMed, Embase, Web of Science, and Scopus. Grey literature was also explored via Google Scholar. All databases were searched from inception to October 23, 2,025, with no language restrictions. Any discrepancies during screening were resolved through consensus with a third reviewer.

Search terms combined MeSH and free-text keywords related to “tuberculosis,” “children,” and “pulmonary function tests,” using Boolean operators (AND/OR). The full search strategies for each database are available in Supplementary Table S1. After removing duplicates, articles were screened by titles and abstracts. Those that appeared potentially relevant were subjected to full-text review for final inclusion. Additionally, reference lists of included studies were examined to find further relevant articles.

### Eligibility criteria

Studies were considered eligible if they met the following PECO framework:

1. Population: Children and adolescents aged ≤18 years with a prior diagnosis of PTB;
2. Exposure: History of PTB with completed treatment;
3. Comparator: Not applicable or healthy/non-TB control groups when available;
4. Outcome: Pulmonary function test (PFT) after completion of anti-TB therapy. Studies were to report values of forced vital capacity (FVC), forced expiratory volume in 1 s (FEV1), FEV1 as a percentage of that predicted (FEV1%), FVC as a percentage of that predicted (FVC%), FEV1/FVC ratio, or any other measurements on PFT. Eligible designs included observational studies—cross-sectional, cohort, or case-control studies.

Exclusion criteria encompassed: (1) studies involving adults or mixed populations where pediatric data were not separately extractable; (2) cases of extrapulmonary TB; (3) absence of PFTs following therapy completion; and (4) reviews, editorials, case reports, or conference abstracts without analysable data. When multiple reports from the same cohort were found, the study with the largest dataset reporting maximum outcomes was selected.

### Data extraction

Two reviewers independently extracted data using a standardised data collection form. The information gathered included study and author details, publication year, country, study design, sample size, demographic info, HIV status, TB diagnostic confirmation, TB treatment specifics, timing and method of PFT evaluation, and key spirometric measures (FEV₁, FVC, and FEV₁/FVC ratio). Disagreements were resolved through discussion with a third reviewer. Corresponding authors were not contacted for missing data, and we did not make assumptions about any missing information.

### Quality assessment

The quality assessment of the included studies was performed using the relevant Joanna Briggs critical appraisal tool for observational studies (12). Each paper was assessed against specific domain questions in the checklist, with responses marked as yes, no, or unclear. Two reviewers independently conducted the quality appraisal, and any disagreements were resolved by arbitration with a senior reviewer.

### Statistical analysis

Statistical analyses were conducted with R software (version 4.2). For studies reporting median and interquartile range, mean and standard deviation values were calculated through established statistical conversions. When studies reported multiple PFT outcomes, we prioritised pre-bronchodilator values and the longest follow-up period. Spirometry results expressed as percentage of predicted values were converted into z-scores using the Global Lung Function Initiative (GLI)-2012 reference equations, consistent with previous review (10, 13). Such data was extracted directly from the earlier meta-analysis (10). Specifically, the GLI-2012 equations provide age-, sex-, height-, and ethnicity-adjusted predicted values and lower limits of normal across the 3–95-year age range. Where ethnicity-specific reference data were not explicitly reported, the default or “other/mixed” GLI category was applied, consistent with prior meta-analytic practice (10, 13). Z-scores were calculated by subtracting the GLI-predicted mean from the observed value and dividing by the GLI-predicted standard deviation. The GLI-2012 reference equations were selected because they are internationally validated across multiple ethnic groups and age categories, including children and adolescents, and are currently recommended for standardised spirometric interpretation (10, 13).

Initially, we combined only post-TB spirometry values to calculate pooled z-scores across studies. Subsequently, in comparative studies, spirometry parameters between TB and control groups were compared to determine effect sizes, expressed as Hedges’ g with 95% confidence intervals (CIs). All pooled estimates were generated with a random-effects model (DerSimonian–Laird method) to account for clinical heterogeneity. Heterogeneity was quantified using the I^2^ statistic, with <50% indicating low and >75% indicating high heterogeneity. Sensitivity analyses involved sequentially excluding individual studies to evaluate the robustness of pooled results. Publication bias was assessed using Egger's test. Subgroup analysis was conducted for post-TB spirometry values based on location, study design, age of participants (>10 years or <10 years), HIV positivity rate, and microbiological confirmation rate.

## Results

### Study selection

**Figure 1:**
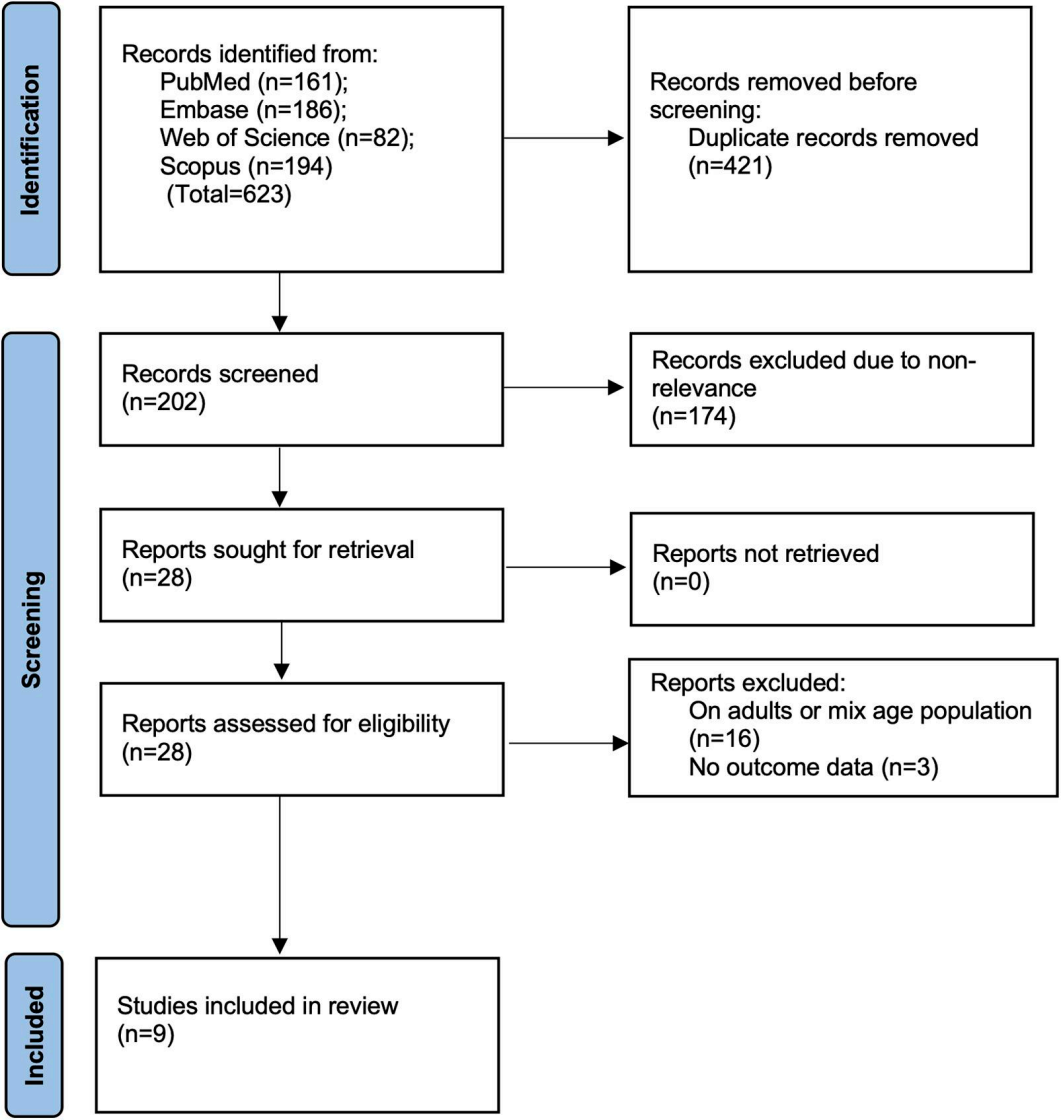
PRISMA flowchart.

A total of 623 records were identified through systematic database searches. After removing 421 duplicates, 202 unique studies were screened based on title and abstract. Of these, 174 did not meet the eligibility criteria and were excluded. The remaining 28 articles underwent full-text review. Following detailed evaluation, nine studies (14–22) met all the predefined inclusion criteria and were included in the final analysis (Figure 1). The main reasons for exclusion included studies focused only on adult or mixed-age populations (*n* = 16) and those lacking extractable data on post-treatment PFT outcomes (*n* = 3).

### Characteristics of included studies

The studies, published between 2019 and 2025, involved 875 children with a history of PTB and, where applicable, 322 controls (Table 1). Study designs included four prospective cohort studies, three cross-sectional studies, and one retrospective cohort. Most studies were conducted in sub-Saharan Africa (South Africa = 5, Gambia = 1, Zimbabwe = 1, Uganda = 1) with one from South Korea. The age of participating children ranged from 5 to 16 years, with male participants making up 38%–74%. The interval between completing anti-TB therapy and PFT ranged from 6 months to over 2 years, depending on the study. Bacteriological confirmation among PTB cases varied from 13.7% to 100%. HIV co-infection was reported in 0%–100% of participants. Most studies did not specify treatment regimens; when they did, the regimens were based on national guidelines. Four studies (15, 18, 19, 21) included a control group of which one reported age-matching. Across studies, one of the following parameters—FEV₁, FVC, or FEV1/FVC ratio—was consistently reported as the main spirometric outcome, except in the case of Martinez et al (20). Their study found that children diagnosed with PTB between 0 and 1 year of age had a shorter time to reach peak tidal expiratory flow relative to total expiratory time and higher fractional exhaled nitric oxide at 5 years. Conversely, children diagnosed with PTB between 1 and 4 years of age exhibited impaired tidal volume and a longer time to peak tidal expiratory flow over total expiratory time at age 5. Because their data were not compatible with other studies for a meta-analysis, it was excluded from the quantitative synthesis. Data conversion from percentage predicted values to z-score was required for one study (16), and such data was extracted from the previous review (10). Another study by Gray et al. (22) did not provide numerical data suitable for meta-analysis. They reported that patients with a chest radiograph suggestive of PTB had lower adjusted z-FEV1 over 12 months (−0.73, 95% CI−1.11,−0.34; *p* < 0.01) and z-FVC (−0.79, 95% CI−1.23,−0.35; *p* < 0.01), compared to patients without such radiographic findings.

**Table 1:** Details of included studies.

Study	Country	Study design	Sample size (with PTB)	Age (years)	Males (%)	Sample size (Control)	Age (years)	Males (%)	Bacteriological confirmation rate for PTB cases (%)	Non-bacteriological diagnosis of PTB	Primary disease domain of included study	HIV status of children with PTB (%)	Time between PTB treatment to PFT
Sovershaeva (14)	Zimbabwe	Cross-sectional	57	15 [12–18]	74	NR	NR	NR	NR	Participant questionnaires	HIV	100	Unspecified
Lee (16)	South Korea	Retrospective cohort	42	11.9 ± 5.6	NR	NR	NR	NR	59.5	Extracted hospital records; Mantoux skin test	Bronchiectasis	Unspecified	Unspecified
Githinji (17)	South Africa	Prospective cohort	305	12 (1.6)	NR	NR	NR	NR	NR	Extracted hospital records and validated study questionnaires	HIV	100	≥24 months
Nkereuwem (21)	Gambia	Cross-sectional	68	8.9 (7.2–11.2)	52.9	91 (age matched)	11.5 (8–13.7)	62.6	35.3	At least two from SS, suggestive CXR, positive response to TB treatment or PE to TB	PTB	13.2	Median: 19.2 (IQR: 10.2–44.4) months
Martinez (20)	South Africa	Prospective cohort	95	5	51.5	NR	NR	NR	13.7	At least two from SS, suggestive CXR or PE to TB	PTB	0	≥6 months, except in cases of drug resistance
van der Zalm (15)	South Africa	Prospective cohort	50	16.2 ± 2	38	50 (house-hold control)	13.4 + 2.5	56	100	–	PTB	10	12 months
Gray (22)	South Africa	Prospective cohort	139	9.8 (7.4, 11.5) for both study and control	NR	30 (non-TB LRTI)	NR	NR	50	SS and one of the following household contact of TB; unexplained weight loss or failure to thrive; positive tuberculin skin test, and suggestive CXR.	PTB	NR	12 months
Becker (19)	Uganda	Cross-sectional	73	9[7–12]	60	49 (house-hold control)	11[8–13]	59	23	SS, contact history, and CXR	PTB	90	Median 1,001 days
Courtney (18)	South Africa	Prospective cohort	46	6[5–9]	54	33 (healthy sibling)	7[5–9]	48	39.1	SS and CXR	PTB	9	52 weeks

PTB, pulmonary tuberculosis; NR, not reported; PFT, pulmonary function test; LRTI, lower respiratory tract infection; CXR, chest radiograph; PE, prior exposure; SS, Suggestive symptoms.

### Post-TB PFT values only

**Figure 2:**
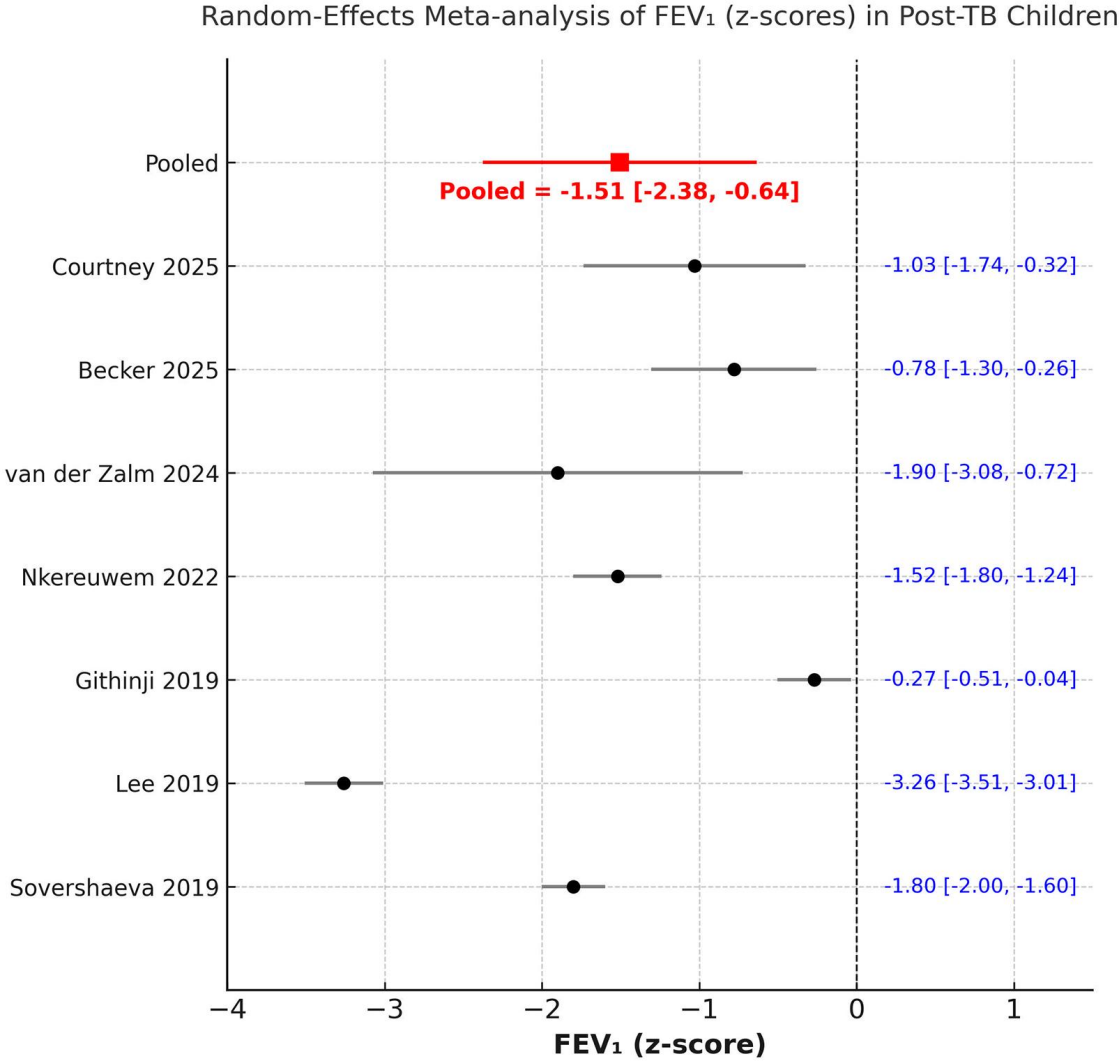
Meta-analysis of FEV_1_ z scores from studies reporting post-PTB PFT data.

**Figure 3:**
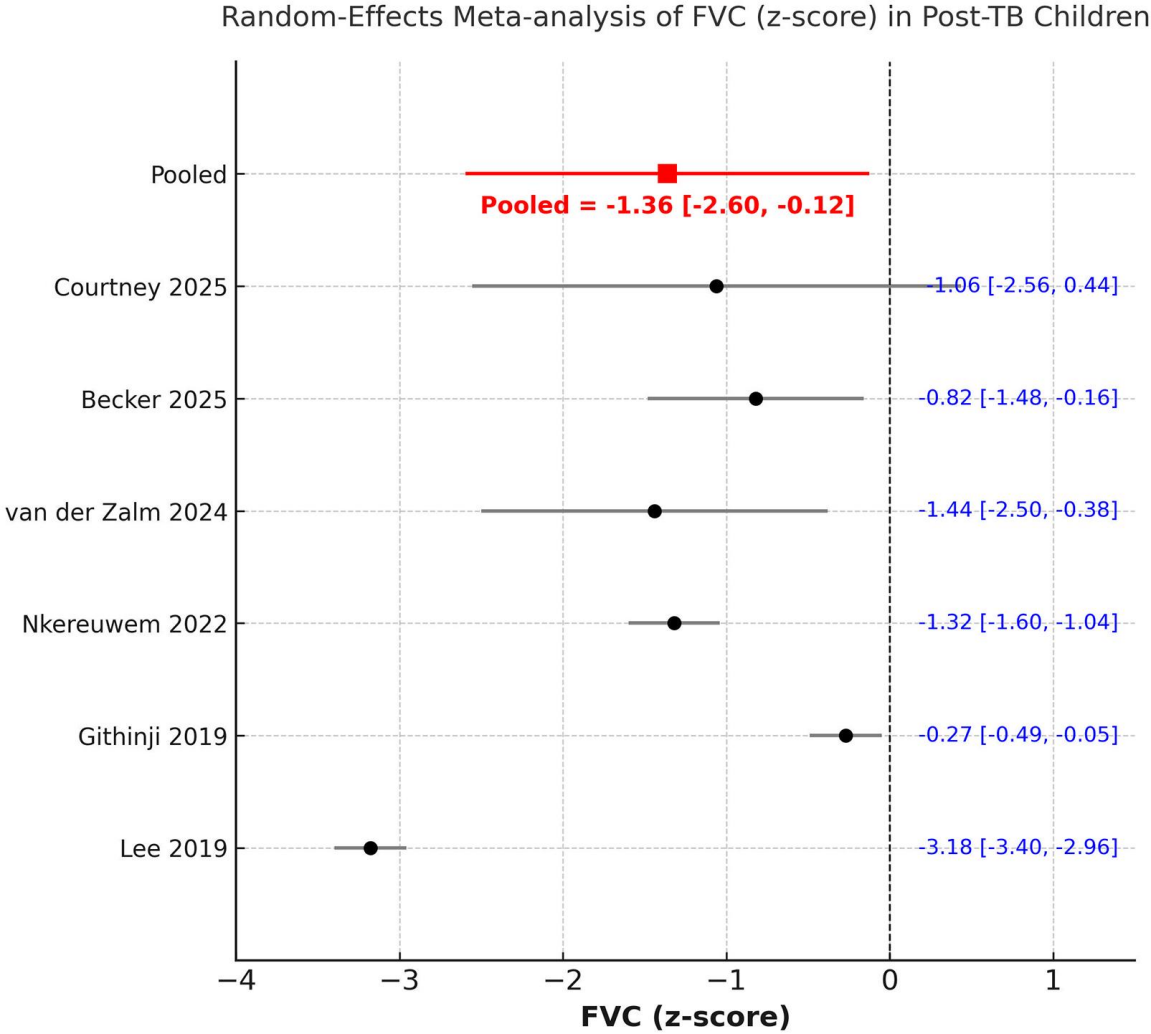
Meta-analysis of FVC z scores from studies reporting post-PTB PFT data.

**Figure 4:**
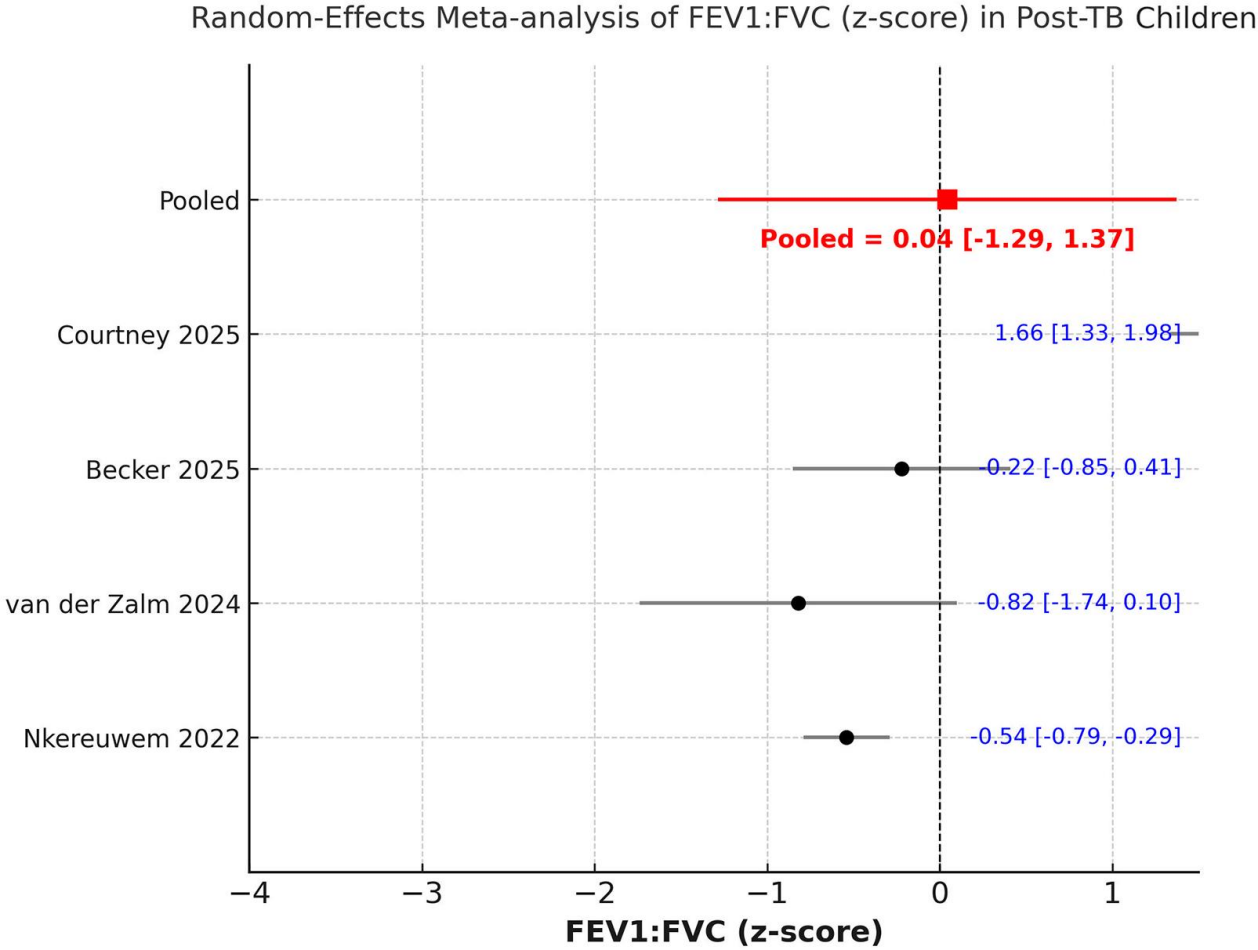
Meta-analysis of FEV1/FVC ratio z scores from studies reporting post-PTB PFT data.

We could include seven studies in the analysis of post-PTB PFT values. A random-effects meta-analysis demonstrated a significant reduction in both FEV₁ and FVC following PTB treatment in children. The pooled mean FEV₁ z-score was −1.51 (95% CI −2.38 to −0.64; I^2^ = 98.1%) (Figure 2), while the pooled FVC (z-score) was −1.36 (95% CI −2.60 to −0.12; I^2^ = 98.6%) (Figure 3). The FEV1/FVC ratio (z-score) showed no significant deviation from zero [0.04 (95% CI −1.29 to 1.37); I^2^ = 97.4%], indicating proportional decline of both FEV₁ and FVC and suggesting a predominantly restrictive pattern of pulmonary dysfunction (Figure 4). Sensitivity analysis results are shown in Supplementary Table S2. There was no change in the significance of the results of FEV₁ and FVC on exclusion of any study, but inter-study heterogeneity remained high. Likewise, the results of FEV1/FVC ratio also remained stable on exclusion of majority studies. Egger's test showed non-significant results (*p* > 0.05) indicating no publication bias.

Subgroup analyses indicated that reductions in FEV₁ and FVC were observed across most categories, although substantial variability persisted between studies (Table 2). Geographic segregation showed marked between-study variability in African studies. FEV1 and FVC reduction was found to be marginally non-significant in cohort studies, but heterogeneity persisted in both cohort and cross-sectional studies. Age-stratified analyses suggested consistent reduction in FEV1 in studies with mean age of <10 years and >10years but FVC reduction was marginally non-significant for FVC. Studies with HIV positivity <15% showed reduced heterogeneity for FEV1 and FVC analysis while studies with HIV positivity rate 90%–100% showed marginally non-significant results for all analyses. Results based on microbiological confirmation rates (>50% and <50%) were also consistent with the primary analysis.

**Table 2:** Subgroup analysis results of post-treatment data.

Variable	Category	Number of studies	Effect size (95% CI)	I^2^ (%)
FEV1				
Location	Africa	6	−1.18 (−1.84, −0.53)	95.2
	Asia	1	−3.26 (−3.51, −3.01)	0
Study design	Cross-sectional	3	−1.43 (−1.89, −0.97)	85.1
	Cohort	4	−1.61 (−3.51, 0.29)	99
Age category	>10	4	−1.80 (−3.19, −0.42)	99
	<10	3	−1.16 (−1.67, −0.64)	70
HIV percentage	90%–100%	3	−0.95 (−2.07, 0.16)	97.9
	<15%	3	−1.46 (−1.75, −1.18)	6.2
Bacteriological confirmation rates	>50%	2	−2.71 (−4.02, −1.40)	79
	<50%	3	−1.16 (−1.67, −0.64)	70
FVC				
Location	Africa	5	−0.93 (−1.57, −0.29)	88.8
	Asia	1	−3.18 (−3.40, −2.96)	0
Study design	Cross-sectional	2	−1.16 (−1.62, −0.71)	46.5
	Cohort	4	−1.50 (−3.48, 0.47)	99.1
Age category	>10	3	−1.63 (−3.92, 0.65)	99.4
	<10	3	−1.24 (−1.49, −0.98)	0
HIV percentage	90%–100%	2	−0.45 (−0.96, 0.05)	58.4
	<15%	3	−1.32 (−1.59, −1.05)	0
Bacteriological confirmation rates	>50%	2	−2.39 (−4.09, −0.69)	89.9
	<50%	3	−1.24 (−1.49, −0.98)	0
FEV1/FVC ratio				
Location	Africa	4	0.04 (−1.29, 1.37)	97.4
Study design	Cross-sectional	2	−0.50 (−0.73, −0.26)	0
	Cohort	2	0.46 (−1.97, 2.89)	96
Age category	>10	1	−0.82 (−1.74, 0.10)	0
	<10	3	0.31 (−1.26, 1.87)	98.2
HIV percentage	90%–100%	1	−0.22 (−0.85, 0.41)	100
	<15%	3	0.12 (−1.58, 1.82)	98.3
Bacteriological confirmation rates	>50%	1	−0.82 (−1.74, 0.10)	0
	<50%	3	0.31 (−1.26, 1.87)	98.2

FVC, forced vital capacity; FEV1, forced expiratory volume in 1s; HIV, human immunodeficiency virus; CI, confidence intervals.

### Comparing TB vs. controls

**Figure 5:**
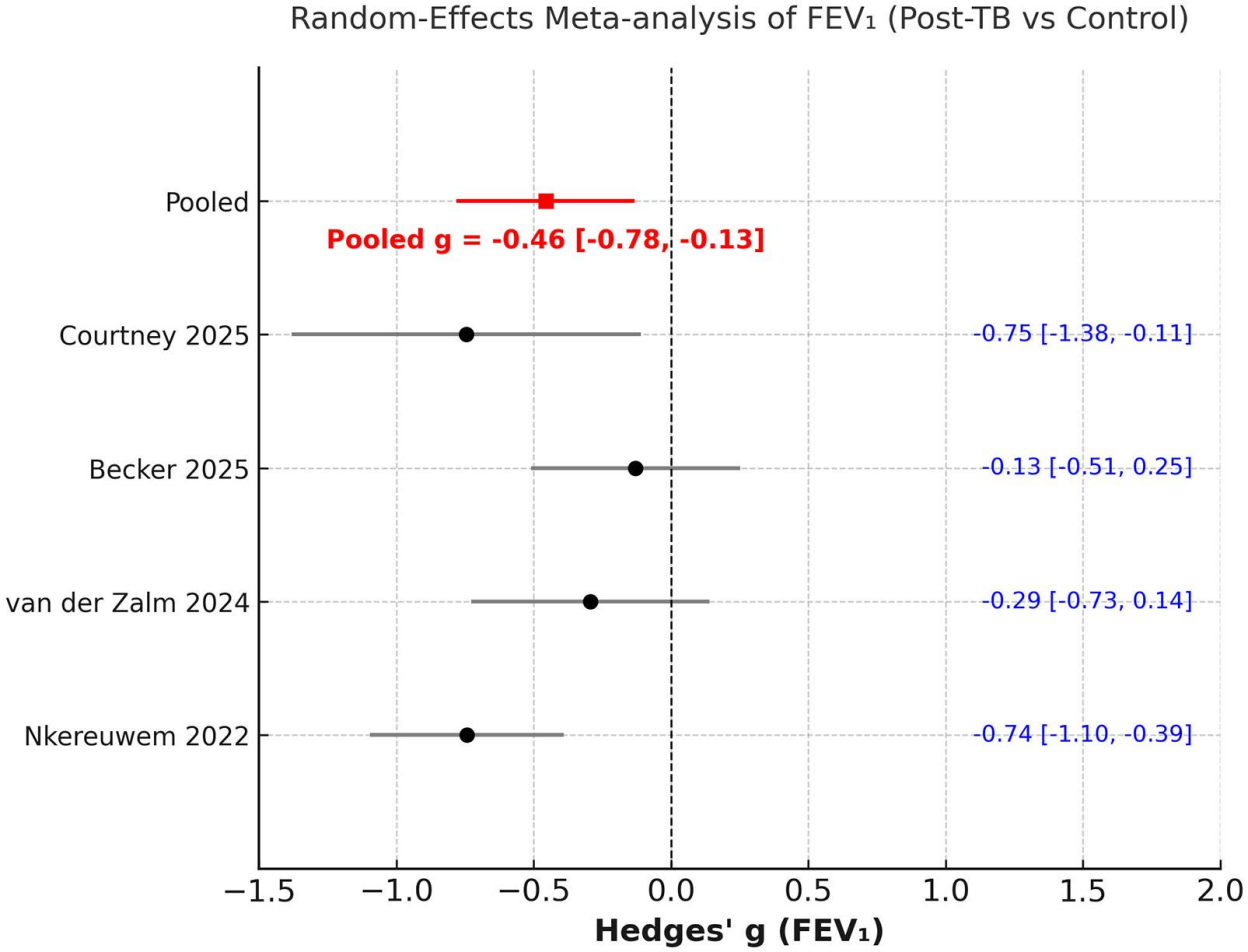
Meta-analysis of comparison of FEV_1_ z scores between post-PTB and controls.

A random-effects meta-analysis was performed using data from four studies to compare PFT parameters between children with a history of PTB and controls. The pooled standardized mean difference (Hedges' g) for FEV₁ was −0.46 (95% CI −0.78 to −0.13), indicating a significant reduction in expiratory function among children previously treated for PTB compared with controls. Between-study heterogeneity was moderate (I^2^ = 55.3%) (Figure 5). Sensitivity analyses, conducted by sequential exclusion of each study, did not show any change in direction of the effect size (Supplementary Table S3).

**Figure 6:**
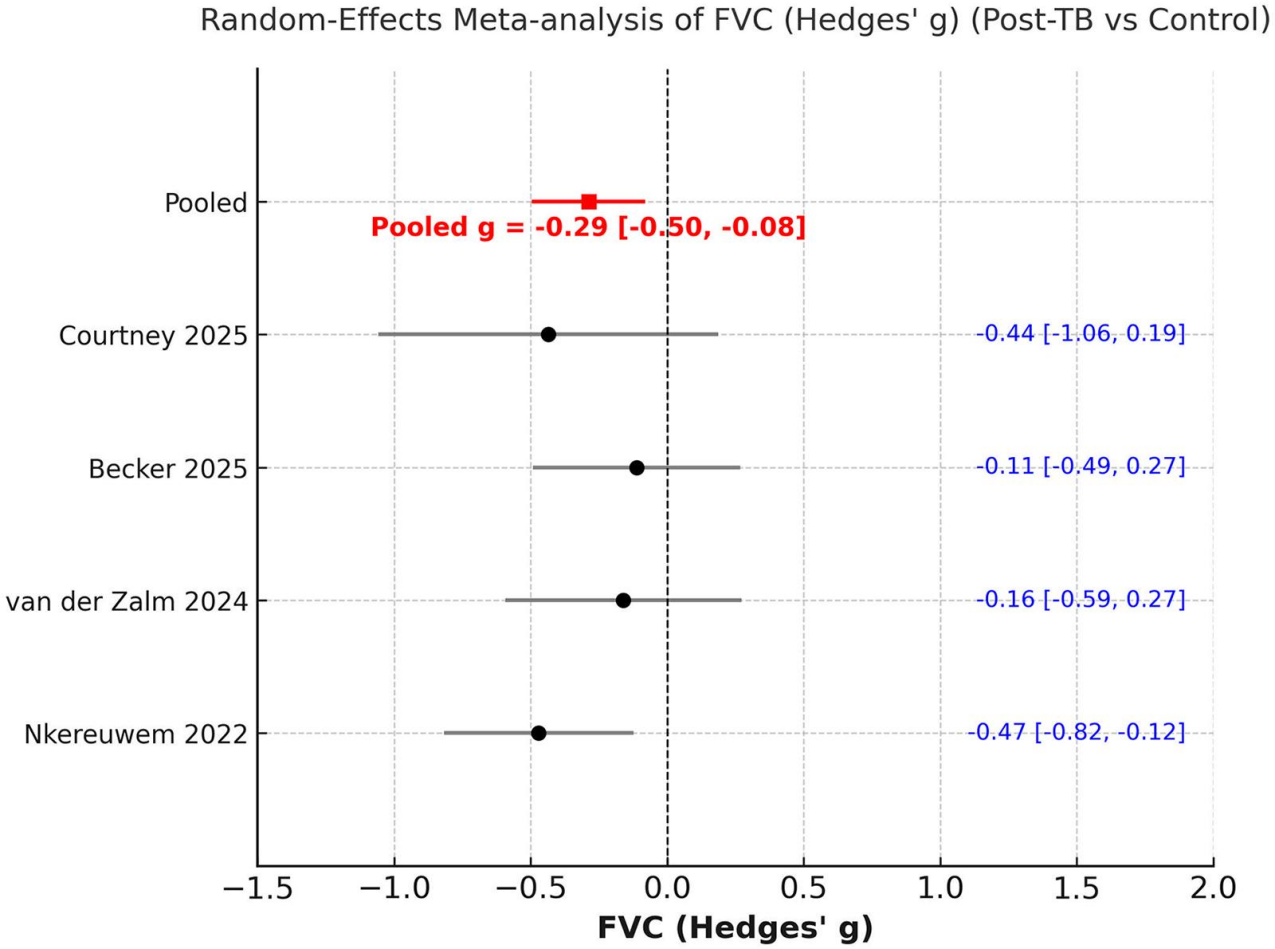
Meta-analysis of comparison of FVC z scores between post-PTB and controls.

The meta-analysis also indicated a significantly reduced FVC in children previously treated for PTB as compared to controls [Hedges' g: −0.29 (95% CI −0.50 to −0.08)]. Heterogeneity was high (I^2^ ≈ 70%) (Figure 6). Leave-one-out analyses showed that exclusion of any single study did not alter the significance of the results, except on exclusion of Nkereuwem et al (21) (Supplementary Table S3).

**Figure 7:**
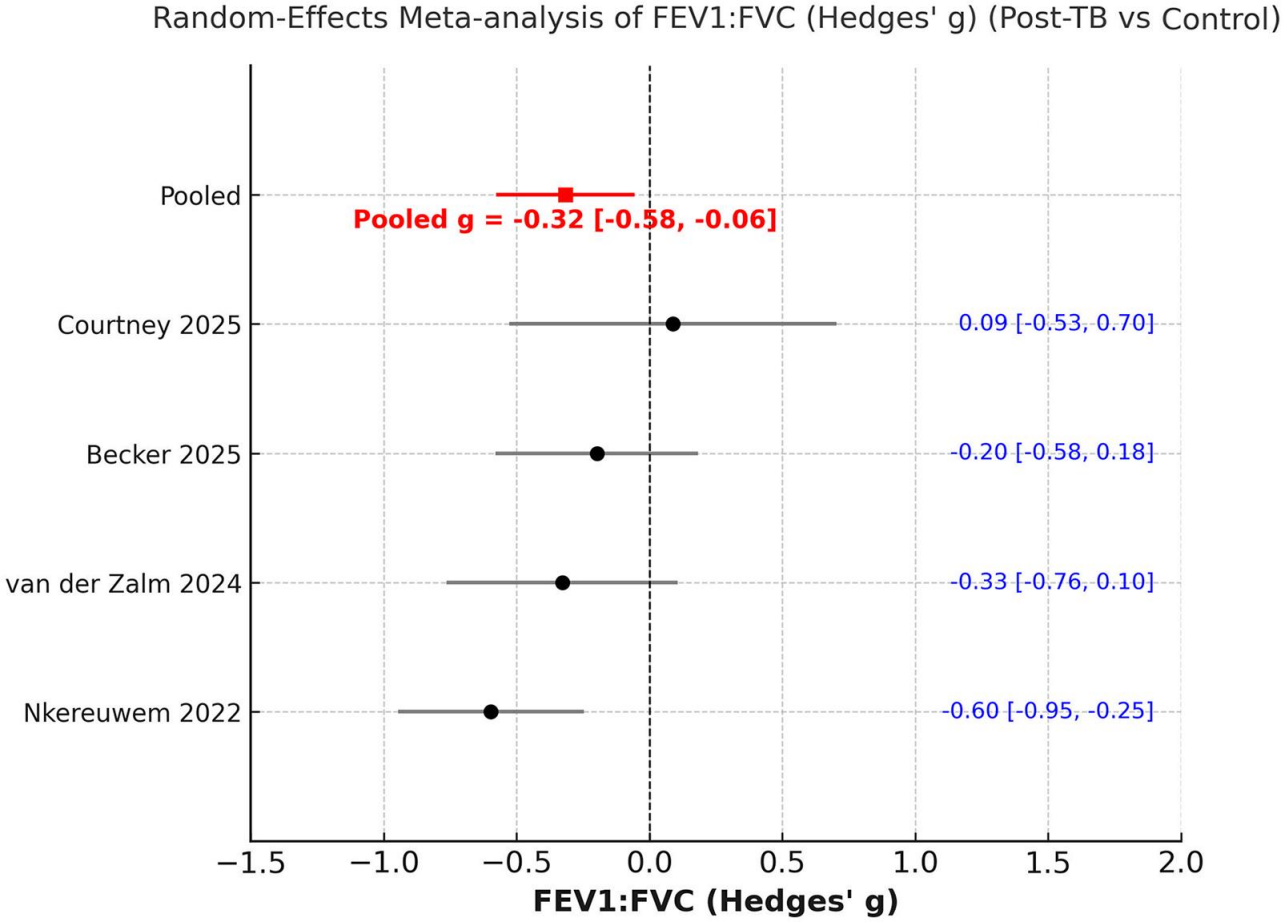
Meta-analysis of comparison of FEV1/FVC ratio z scores between post-PTB and controls.

The pooled Hedges' g for the FEV1/FVC ratio was −0.32 (95% CI −0.58 to −0.06), showing marginally reduced ratio in children previously treated for PTB as compared to control (Figure 7). Heterogeneity remained moderate-to-high (I^2^ ≈ 60%), and sensitivity analyses indicated unstable results with loss of significance on exclusion of most studies (Supplementary Table S3). Egger's test did not reveal any publication bias in the above analyses.

### Risk of bias analysis

Quality assessment of included studies is shown in Supplementary Table S4. A major concern across studies was residual confounding. Socioeconomic status, malnutrition, household air pollution, passive smoke exposure, and HIV infection are well-recognised determinants of impaired lung development in children; however, these factors were inconsistently reported and rarely adjusted for in multivariable analyses. Additionally, most studies did not account for baseline lung function prior to PTB diagnosis. Several studies also had risk of bias pertaining to assessment of exposure (PTB) as bacteriological confirmation was not used in all studies.

## Discussion

This systematic review and meta-analysis offer the most current synthesis of evidence regarding pulmonary function outcomes in pediatric patients following successful treatment for PTB. Data from nine recent studies, involving a total of 875 children post-PTB treatment, were analysed to demonstrate that lung function remains significantly impaired several months after the completion of therapy. Specifically, the mean FEV₁ and FVC z-scores were approximately 1.5 standard deviations below predicted normative values. In the case-control meta-analysis, children who had previously undergone treatment for PTB showed significantly lower FEV₁ (Hedges' g = −0.46) and modestly reduced FVC (Hedges' g = −0.29) compared to healthy controls, while the FEV1/FVC ratio was relatively preserved.

A key insight from this review is the significant under-representation of pediatric and adolescent groups in studies of PTB-related respiratory complications over the last twenty years. Most published research focuses solely on adults, highlighting a critical gap in understanding outcomes for younger populations. This exclusion constrains our knowledge of the full range of disease effects, as evidenced by the limited number of pediatric studies available. Our findings differ somewhat from those in the recent large meta-analysis by Ratnakumar et al. (23), which mainly involved adult participants from 22 countries, totalling over 75,000 individuals. Analysing data from 19 studies, prior PTB was associated with significant declines across all spirometric measures, with average decreases of −0.41 L in FEV₁ and −0.25 L in FVC. The pooled standardised mean differences were −0.44 for FEV₁% and −0.33 for FVC%. In adults, the pattern suggested mixed ventilatory issues, mainly airflow obstruction, indicated by a lower FEV₁/FVC ratio. In children, however, the meta-analysis showed that despite substantial reductions in FEV₁ and FVC z-scores (around ≈ −1.5), the FEV1/FVC ratio was mostly maintained. In case-control studies, the standardised differences were smaller (FEV₁ = −0.46; FVC = −0.29; FEV1/FVC ratio = −0.32), with the effect sizes for FEV₁ and FVC remaining significant after sensitivity analysis, but not for FEV1/FVC ratio. This suggests a less severe obstructive pattern and more of a restrictive or growth-related lung impairment in children.

It is also important to compare our findings with the previous meta-analysis on this topic. Our results both support and expand on those reported by Lew et al. (10), who conducted the first systematic review and meta-analysis on lung function in children after PTB. This earlier study pooled data from just five studies (*n* = 567) and found significant reductions in both FEV₁ (−1.53 z, 95% CI −2.65 to −0.41) and FVC (−1.93 z, 95% CI −3.35 to −0.50) after treatment. In contrast, our updated review includes eight studies (*n* = 736), with three additional cohorts published between 2023 and 2025. An important advancement in our review is its broader analytical scope. While Lew et al. (10) focused only on post-treatment cohorts, our study conducted two complementary meta-analyses: one evaluating absolute spirometric deficits after PTB, and another comparing children previously treated for PTB with healthy peers. This dual approach validates lung function impairment after PTB treatment and assesses its extent relative to unaffected groups.

Residual confounding remains an important consideration when interpreting our findings. Children with a history of PTB face various confounding factors that make it challenging to attribute spirometric deficits solely to the PTB episode. For instance, nutritional deficiencies, recurrent respiratory illnesses, household smoke exposure, air pollution, passive smoking and HIV coinfection are known to influence lung development and can independently affect PFT in children (4, 24, 25). The majority of studies included in this review did not fully account for these factors or lacked appropriate control groups matched for socio-environmental risks, which means that some of the observed lung impairment might result from early-life disadvantages rather than direct damage from TB. Secondly, the timing of spirometry testing relative to treatment completion and growth stages also adds heterogeneity: children tested shortly after therapy may still be catching up in lung growth, while those tested later may have encountered additional exposures. A recent study in adults indicates that PFT values decline after 3 years, compared with measurements taken 12 months post-PTB treatment (26). Thirdly, the broad age range of children included in these studies introduces significant heterogeneity. Younger children, especially those under two, are more prone to severe and disseminated TB. Conversely, children aged 2–10 often develop disease mainly involving intrathoracic lymph nodes, with less lung parenchymal involvement. As children progress into adolescence, the disease pattern tends to resemble adult PTB, characterised by higher bacillary loads, substantial lung damage, cavitation, and fibrosis (2, 27). It therefore remains uncertain whether these differing clinical presentations, along with ongoing lung development, lead to different risks or types of respiratory aftereffects post-PTB. Collectively, the high heterogeneity observed in our analyses may partly stem from these major issues. This limitation reduces confidence in attributing the mean deficits solely to PTB; rather, the results should be seen as indicating lung-function impairment associated with post-PTB status in environments with multiple overlapping risks. Future research should focus on using age-, socio-economic-, and environmentally matched control groups, conducting longitudinal follow-ups, and applying thorough adjustments for baseline confounding factors to more accurately determine the true effects attributable to TB in children.

Childhood PTB can lead to long-term respiratory issues by damaging lung tissue, causing airway problems, and disrupting lung growth (28–30). The initial infection typically results in caseating necrosis and cavity formation, which can heal with fibrotic scars and loss of functional alveoli, reducing lung volume in a restrictive pattern. Additionally, airway involvement, including lymph node pressure, bronchial narrowing, and bronchiectasis, contributes to airflow restriction and ventilation heterogeneity (29, 30). In children, these injuries occur during a critical phase of lung development, as alveoli and airways continue to grow into adolescence. Injury at this stage may permanently restrict maximum lung capacity and lower FEV₁ and FVC scores (28).

Several limitations remain in this review. Firstly, the available evidence base remains small and geographically concentrated. Although this updated meta-analysis identified eight studies, the data were still derived predominantly from a limited centres in sub-Saharan Africa, with minimal representation from other regions. As a result, the pooled estimates may not be generalizable to all global pediatric populations affected by PTB. Second, substantial clinical and methodological heterogeneity was observed across studies. The heterogeneity likely reflects variations in study design, age range, disease severity, the interval between treatment completion and spirometry, and the clinical phenotype of TB across the pediatric age spectrum. While subgroup analysis was conducted for post-TB PFT values to explore the source of heterogeneity, the same could not be conducted for case-control data due to limited number of studies. Moreover, most of the results of the subgroup analysis were consistent with the overall analysis and high heterogeneity persisted in most subgroups. While marginal non-significant results were seen in some subgroups, it could be attributed to the small number of studies. The persistent high heterogeneity in subgroups indicates that other factors like interval between treatment completion and spirometry, environmental exposures, and socio-economic factors, which were inconsistently reported and rarely adjusted for, may have caused residual confounding. Also, given the limited number of eligible studies, statistical power to explore heterogeneity through meta-regression was restricted. Third, microbiological confirmation of TB was inconsistent across studies. Several included cohorts diagnosed TB using clinical or radiographic criteria alone, raising the possibility of misclassification or inclusion of non-TB respiratory illnesses. Nevertheless, it must be remembered that globally, only 63% of PTB cases are diagnosed with microbiological confirmation (23). Some studies focused solely on HIV-infected children or patients with post-TB bronchiectasis, which may limit the ability to compare pooled estimates and may exaggerate the observed effects. Fourthly, relying only on spirometry restricts understanding of the full range of post-PTB lung abnormalities. The majority of studies did not include data on lung volumes, diffusion capacity, plethysmography, or imaging, which could help distinguish airway from parenchymal issues. The lack of standardised bronchodilator testing across several studies also hampers comparability, especially when distinguishing between reversible airway obstruction and fixed restrictive defects. Fifthly, there was lack of differentiation between drug-sensitive and drug-resistant TB in the included studies. Given that drug-resistant TB is typically associated with prolonged treatment, higher bacillary burden, delayed microbiological clearance, and more extensive structural lung damage, the absence of stratified analysis may obscure clinically meaningful differences in long-term pulmonary outcomes. This limits the applicability of our pooled estimates to specific treatment subgroups. Sixthly, several studies were cross-sectional, offering only a single post-treatment measurement without baseline or follow-up data. Therefore, a causal relationship between PTB and lung function impairment cannot be fully determined from the current. Publication bias may also influence results, as studies with null or minimal findings are less likely to be published. Finally, although the study protocol was registered with PROSPERO, a comprehensive, formal protocol was not created or published, which may affect transparency and reproducibility. There were also minor deviations from the registered protocol, such as using the Joanna Briggs Institute tool for quality assessment instead of the Newcastle Ottawa Scale listed at registration, and a lack of clarity regarding the use of AI-assisted methods. Nonetheless, these deviations are unlikely to have significantly impacted the overall results of the review.

This review's findings have significant clinical implications for the long-term management of children treated for PTB. Ongoing reductions in lung function, even after microbiological cure, emphasise the importance of systematic respiratory follow-up in pediatric PTB survivors. Incorporating spirometry or other lung function tests into routine post-treatment care can help identify children at risk of chronic respiratory issues early, enabling timely interventions like pulmonary rehabilitation. From a public health standpoint, these results highlight the need to prevent PTB infection during critical periods of lung development, as early injuries may cause irreversible effects and heighten the risk of lifelong respiratory diseases. Enhancing integrated TB and lung health programs within national TB strategies could be essential in reducing the long-term respiratory impacts of childhood PTB.

## Conclusions

This updated systematic review and meta-analysis indicates that children who have completed PTB treatment frequently exhibit FEV₁ and FVC values below age matched reference levels, although estimates vary substantially across studies. Pooled effect sizes should be interpreted as indicative of association rather than precise estimates of magnitude. The notable heterogeneity across studies is a cause of concern and results must be interpreted with caution till further more substantial evidence is available.

## Data Availability

Publicly available datasets were analyzed in this study. This data can be found here: we searched PubMed, Embase, Web of Science, and Scopus up to October 23, 2025.
