# Supplementary material for: Lung function impairment in children post-tuberculosis treatment: a systematic review and meta-analysis
Source: Front Pediatr. 2026 Apr 23;14:1753683. doi: 10.3389/fped.2026.1753683 (PMC13149381; doi:10.3389/fped.2026.1753683)
Supplement: Supplementary file 1 [file Table1.docx]

# Supplementary Table 1. Detailed Search Strategy

| Database | Search Strategy |
| --- | --- |
| PubMed | ("Tuberculosis, Pulmonary"[Mesh] OR "tuberculosis"[Title/Abstract] OR "pulmonary tuberculosis"[Title/Abstract] OR "post tuberculosis"[Title/Abstract] OR "post-TB"[Title/Abstract] OR "post-tuberculosis lung disease"[Title/Abstract] OR "PTLD"[Title/Abstract]) AND ("Child"[Mesh] OR "Infant"[Mesh] OR "Adolescent"[Mesh] OR child[Title/Abstract] OR infant[Title/Abstract] OR adolescent[Title/Abstract] OR pediatric[Title/Abstract] OR paediatric[Title/Abstract]) AND ("Pulmonary Function Tests" OR "Spirometry"[Mesh] OR "Respiratory Function Tests"[Mesh] OR "lung function"[Title/Abstract] OR "pulmonary function"[Title/Abstract] OR spirometry[Title/Abstract] OR "FEV1"[Title/Abstract] OR "FVC"[Title/Abstract] OR "FEV1/FVC"[Title/Abstract] OR "lung capacity"[Title/Abstract]) AND ("Treatment Outcome"[Mesh] OR "therapy" OR "treatment"[Title/Abstract] OR "post-treatment"[Title/Abstract] OR "after treatment"[Title/Abstract]) |
| Embase | ('pulmonary tuberculosis'/exp OR 'tuberculosis'/exp OR 'post tuberculosis':ti,ab OR 'post-TB':ti,ab OR 'post-tuberculosis lung disease':ti,ab OR PTLD:ti,ab) AND ('child'/exp OR 'infant'/exp OR 'adolescent'/exp OR child:ti,ab OR adolescent:ti,ab OR pediatric:ti,ab OR paediatric*:ti,ab) AND ('lung function test'/exp OR 'pulmonary function test'/exp OR 'spirometry'/exp OR spirometr*:ti,ab OR 'FEV1':ti,ab OR 'FVC':ti,ab OR 'FEV1/FVC':ti,ab OR 'respiratory function test':ti,ab OR 'lung capacity':ti,ab) AND ('treatment outcome'/exp OR 'treatment'/exp OR 'therapy'/exp OR 'after treatment':ti,ab OR 'post-treatment':ti,ab) |
| Web of Science | TS = (("pulmonary tuberculosis" OR "post-tuberculosis" OR "post TB" OR "post-tuberculosis lung disease" OR PTLD) AND (child* OR pediatric* OR paediatric OR adolescent OR infant) AND ("pulmonary function" OR "lung function" OR "respiratory function" OR spirometr* OR "FEV1" OR "FVC" OR "FEV1/FVC" OR "lung capacity") AND ("treatment" OR "therapy" OR "treatment outcome" OR "post-treatment" OR "after treatment")) |
| Scopus | TITLE-ABS-KEY ("pulmonary tuberculosis" OR "tuberculosis" OR "post-tuberculosis" OR "post-TB" OR "post-tuberculosis lung disease" OR PTLD) AND TITLE-ABS-KEY (child OR infant OR adolescent OR pediatric OR paediatric) AND TITLE-ABS-KEY ("lung function" OR "pulmonary function" OR "respiratory function" OR spirometr* OR "FEV1" OR "FVC" OR "FEV1/FVC" OR "lung capacity") AND TITLE-ABS-KEY ("treatment" OR "therapy" OR "treatment outcome" OR "post-treatment" OR "after treatment") |
