# Supplementary material for: Lung function impairment in children post-tuberculosis treatment: a systematic review and meta-analysis
Source: Front Pediatr. 2026 Apr 23;14:1753683. doi: 10.3389/fped.2026.1753683 (PMC13149381; doi:10.3389/fped.2026.1753683)
Supplement: Supplementary file 2 [file Table2.docx]

Supplementary Table 2: Sensitivity analysis results

FEV_1_

| **Excluded study** | **Pooled mean** | **95% CI lower** | **95% CI upper** | **I² (%)** |
| --- | --- | --- | --- | --- |
| Sovershaeva 2019 | -1.46 | -2.62 | -0.30 | 98.36 |
| Lee 2019 | -1.18 | -1.84 | -0.53 | 95.18 |
| Githinji 2019 | -1.73 | -2.50 | -0.96 | 96.44 |
| Nkereuwem 2022 | -1.50 | -2.57 | -0.44 | 98.37 |
| van der Zalm 2024 | -1.45 | -2.39 | -0.52 | 98.38 |
| Becker 2025 | -1.63 | -2.59 | -0.67 | 98.32 |
| Courtney 2025 | -1.58 | -2.54 | -0.63 | 98.36 |

FVC

| **Excluded study** | **Pooled mean** | **95% CI lower** | **95% CI upper** | **I² (%)** |
| --- | --- | --- | --- | --- |
| Lee 2019 | -0.93 | -1.57 | -0.29 | 88.81 |
| Githinji 2019 | -1.61 | -2.78 | -0.44 | 96.99 |
| Nkereuwem 2022 | -1.36 | -3.03 | 0.30 | 98.84 |
| van der Zalm 2024 | -1.35 | -2.71 | 0.02 | 98.85 |
| Becker 2025 | -1.47 | -2.87 | -0.07 | 98.83 |
| Courtney 2025 | -1.41 | -2.76 | -0.06 | 98.85 |

FEV_1_:FVC

| **Excluded study** | **Pooled mean** | **95% CI lower** | **95% CI upper** | **I² (%)** |
| --- | --- | --- | --- | --- |
| Nkereuwem 2022 | 0.24 | -1.38 | 1.86 | 95.55 |
| van der Zalm 2024 | 0.31 | -1.26 | 1.87 | 98.23 |
| Becker 2025 | 0.12 | -1.58 | 1.82 | 98.27 |
| Courtney 2025 | -0.52 | -0.74 | -0.29 | 0.00 |

CI, Confidence intervals
