# Supplementary material for: Lung function impairment in children post-tuberculosis treatment: a systematic review and meta-analysis
Source: Front Pediatr. 2026 Apr 23;14:1753683. doi: 10.3389/fped.2026.1753683 (PMC13149381; doi:10.3389/fped.2026.1753683)
Supplement: Supplementary file 3 [file Table3.docx]

Supplementary Table 3: Sensitivity analysis for case-control data

FEV_1_

| **Excluded study** | **Pooled g** | **95% CI lower** | **95% CI upper** | **I^2^ (%)** |
| --- | --- | --- | --- | --- |
| Nkereuwem 2022 | -0.31 | -0.62 | 0.00 | 24.93 |
| van der Zalm 2024 | -0.52 | -0.96 | -0.08 | 66.96 |
| Becker 2025 | -0.59 | -0.89 | -0.28 | 27.24 |
| Courtney 2025 | -0.40 | -0.78 | -0.02 | 65.45 |

FVC

| **Excluded study** | **Pooled g** | **95% CI lower** | **95% CI upper** | **I^2^ (%)** |
| --- | --- | --- | --- | --- |
| Nkereuwem 2022 | -0.19 | -0.45 | 0.07 | 0.00 |
| van der Zalm 2024 | -0.33 | -0.56 | -0.09 | 0.02 |
| Becker 2025 | -0.36 | -0.61 | -0.11 | 0.00 |
| Courtney 2025 | -0.27 | -0.50 | -0.04 | 8.84 |

FEV_1_: FVC

| **Excluded study** | **Pooled g** | **95% CI lower** | **95% CI upper** | **I^2^ (%)** |
| --- | --- | --- | --- | --- |
| Nkereuwem 2022 | -0.19 | -0.45 | 0.07 | 0.00 |
| van der Zalm 2024 | -0.29 | -0.66 | 0.08 | 55.12 |
| Becker 2025 | -0.35 | -0.70 | 0.01 | 46.36 |
| Courtney 2025 | -0.39 | -0.63 | -0.15 | 16.42 |
