# Supplementary material for: Lung function impairment in children post-tuberculosis treatment: a systematic review and meta-analysis
Source: Front Pediatr. 2026 Apr 23;14:1753683. doi: 10.3389/fped.2026.1753683 (PMC13149381; doi:10.3389/fped.2026.1753683)
Supplement: Supplementary file 4 [file Table4.docx]

Supplementary Table 4: Risk of bias analysis

| Questions | Sovershaeva 2019 | Lee 2019 | Githinji 2019 | Nkereuwem 2022 | Martinez 2023 | van der Zalm 2024 | Becker 2025 | Courtney 2025 |
| --- | --- | --- | --- | --- | --- | --- | --- | --- |
| Were the criteria for inclusion in the sample clearly defined? | Y | Y | Y | Y | Y | Y | Y | Y |
| Were the study subjects and the setting described in detail? | Y | Y | Y | Y | Y | Y | Y | Y |
| Was the exposure measured in a valid and reliable way? | N | N | N | N | N | Y | N | N |
| Were objective, standard criteria used for measurement of the condition? | N | Y | Y | Y | Y | Y | Y | Y |
| Were confounding factors identified? | N | N | N | N | N | N | N | N |
| Were strategies to deal with confounding factors stated? | N | N | N | N | N | N | N | N |
| Were the outcomes measured in a valid and reliable way? | Y | Y | Y | Y | Y | Y | Y | Y |
| Was appropriate statistical analysis used? | Y | Y | Y | Y | Y | Y | Y | Y |

Y, Yes; N, No
